# Novel Intraoperative Applications of Fluorescence Imaging Using Indocyanine Green in Pediatric Urology

**DOI:** 10.1007/s11934-025-01256-6

**Published:** 2025-02-05

**Authors:** Albert S. T. Lee, Ching Man Carmen Tong

**Affiliations:** 1 Division of Urology, Texas Children’s Hospital, Houston, USA; 2 Department of Urology, Baylor College of Medicine, Houston, USA; 3 Department of Urology, University of Alabama at Birmingham, 1600 7th Avenue South Suite 318, Lowder Building, Birmingham, AL 35233 USA

**Keywords:** Fluorescence imaging, Pediatric urology, Indocyanine green, Robotics, Minimally invasive surgery

## Abstract

**Purpose of Review:**

Near-infrared fluorescence imaging (NIRF) with the use of indocyanine green (ICG) has been recently adopted in pediatric urology after its well-published use in the adult population. As a powerful tool that can help delineate complex anatomy and congenital anomalies, we discuss the various applications of this imaging in minimally invasive and open surgery in pediatric urology.

**Recent Findings:**

The most reported applications of ICG in pediatric urology are within minimally invasive surgery, particularly varicoceles, renal surgery such as nephrectomies and renal tumor excision, mimicking its use in adult urology. ICG has also been applied to reconstructive urology such as ureteral reconstruction, hypospadias repair and bladder exstrophy. Despite its safety and more widespread use in pediatric surgery, all published studies in pediatric urology to date have been limited to small and single-center experiences, reflecting the novel nature of this technology in this field.

**Summary:**

ICG has been shown to be safe and effective in children, particularly in those with complex anatomy and in technically challenging surgeries. Future studies should focus on standardized protocols for children and multi-center comparative studies.

## Introduction

In the last several decades, technological advancements in surgical techniques and imaging, such as magnification with microscopy and loupes, robotic surgery and precise instrumentation, have improved operative outcomes and success rates. Pediatric surgeries in particular have benefited from these improvements, where small anatomic details, aberrant blood supply and ablated tissue planes in complex congenital conditions are commonly encountered intraoperatively.

One such imaging technique is the use of Indocyanine green (ICG), a type of fluorescence imaging that can help identify anatomic structures, visualize blood supply as well as tissue perfusion. The Food and Drug Administration (FDA) approved its use for health applications in the 1950s [1]. Early adopters were cardiac and liver specialists who used ICG fluorescence to assess cardiac function and liver blood flow, but now its use has expanded into ophthalmology, colorectal surgery, and of course, urology [2, 3]. With its low cost and easy learning curve, ICG imaging has emerged as a powerful tool to enhance precision and safety in surgery with complex anatomy. Herein, the authors review the safety and benefits of this technology in children and the published innovative applications within pediatric urology.

### Mechanism of Action

Indocyanine green (ICG) is a water-soluble fluorochrome that generates fluorescence when stimulated by near-infrared (NIR) light [4]. After the fluorochrome absorbs light energy and returns from an excited state back to neutral, it emits energy in the form of photons at a wavelength of approximately 800 nanometers [5]. Initially created for NIR photography by Kodak Research Laboratories in 1955, it has since been applied to clinical practice since 1956^2^.

ICG does not have any known metabolites. It bounds to protein almost immediately after injection, thereby confining it to the intravascular space [6]. It has a half-life of less than 240 s and is excreted entirely into bile by the liver. It has very minimal to no uptake by lungs, cerebrospinal system or peripheral vasculature. Visualization of anatomy is typically achieved within 60 s. Dosage of ICG varies widely in surgical literature, but most doses range from 0.01 mg/kg to a maximum dose of 2 mg/kg in children [7]. The ICG vial comes as a powder in 5 mg/mL container, and should be dissolved with sterile water just prior to injection. The reconstituted solution is valid for 6 h only; after this the molecule becomes unstable and not suitable for intraoperative use [8]. ICG, as described later, can be injected intravenously as a bolus or peripherally for lymphatic and tissue mapping.

### Advantages

A major advantage of using ICG is the lack of ionizing radiation, thereby adhering to the principles of ALARA (‘as low as reasonably achievable’) [9]. This is particularly important in children whose tissues are more radiosensitive and are likely to receive more ionizing studies throughout their lifetime. ICG also produces high-quality spatial resolution and it is easy to administer [10, 11]. With its high water solubility and rapid biliary excretion, it is particularly useful for visualizing tissue perfusion [12]. There is also wide availability and variety of camera systems with different brands. Near-infrared fluorescence imaging is integrated on the Intuitive Da Vinci Surgical Systems with Firefly technology [13].

ICG is known for its safety profile. Even in infants, toxicity adverse effects are very rare, primarily relating to allergic reactions to iodinated contrast. It is quick to fluoresce after administration with minimal intraoperative wait time, unlike other intravenous fluorescence agents. The learning curve is easy, since it is easy to reconstitute and administer.

### Disadvantages

With a short half-life of around 3 min, ICG does require redosing, typically after 10–15 min for repeat fluorescence. There have not been any standardized dosing protocols for ICG, and it is unclear if dosing should be based primarily on weight or age. Its use is contraindicated in patients with known allergies to iodinated contrasts; case reports of anaphylaxis have been reported in the past [14]. It has been suggested that ICG should be used with caution in patients with renal failure or on dialysis. Finally, for patients in need of iodine-uptake studies, it is recommended that these exams be delayed at least 1 week following ICG use, as ICG may affect the iodine-binding function of the thyroid [13].

From a technical standpoint, in cases where ICG imaging is being used to quantify perfusion, there can be a significant learning curve to using the ICG quantification software intraoperatively. As this is still a new technology in pediatric surgery, there are no defined cutoff values to correlate perfusion with improved outcomes and decreased stricture rates. In operative fields that are smaller and restricted, if multiple doses of ICG were used, ICG may be retained in congested tissue which lead to interpretation of falsely positive uptake [15].

### Applications in Pediatric Urology

One of the most described utilizations of ICG in pediatric urology is in minimally invasive surgical procedures (MIS). As mentioned previously, all Intuitive da Vinci systems that are Xi and newer have ICG technology integrated within the platform, thereby negating the need for a specific camera, light sources and optic systems to visualize ICG fluorescence intraoperatively [16]. Some of the procedures done with MIS and ICG in pediatric urology are listed below:

### Varicocelectomy

The application of ICG is perhaps most robustly described in varicocele repair. One of the most common complications of laparoscopic varicocelectomy is the formation of hydroceles, which can occur in up to 30% of cases. First described by Esposito and colleagues in 2019, lymphatic sparing Palomo technique can be easily performed when 0.5 mg was injected directly into the body of the testis. Using near-infrared and standard white light mode, lymphatic vessels fluoresced and were spared during the procedure [17]. It is hypothesized that ICG allows for the identification of lymphatics which can help spare lymphatic disruption and decrease the risks of hydrocele formation. Esposito et al. later reported the safety and feasibility of using ICG during MIS varicocelectomy in 41 patients. They again injected the ICG in the testes and did not have any testicular atrophy or hydrocele at the follow up [18].

#### Nephrectomy

ICG enables better visualization of the various anatomical structures such as vessel identification and differentiating areas of ischemia and perfusion. Esposito et al. reported their results of 3 laparoscopic nephrectomies and 9 partial nephrectomies for non-tumor purposes. They injected ICG after division of Gerota’s fascia and found no differences in the results in nephrectomies when compared to one done without ICG [18].

#### Hemi-Nephrectomy

In those that underwent partial nephrectomies for non-functioning/poorly functioning renal moiety, Esposito et al. demonstrated lower median operative time and lower postoperative complication rate in their series of 12 patients that underwent laparoscopic partial nephrectomy when compared to 10 patients that underwent the same procedure but without ICG [19]. Similarly, Herz et al. used ICG during hemi-nephrectomies as a method for selective arterial mapping to avoid any injuries to the moiety that’s not being removed. They observed no complications to the remaining moiety in the 6 patients that underwent hemi-nephrectomies with ICG [20].

#### Partial Nephrectomy for Kidney Tumor

ICG can also help aid in the Identification of tumors through its “second window” property. Due to the tumor’s leaky microvasculature and inefficient lymphatic drainage, ICG injected the day before surgery would bind to plasma proteins and form macromolecular complexes retained in the tumor. Abdelhafeez et al. aimed to utilize this property to help identify renal tumors in 8 pediatric patients with renal tumors in 12 kidneys. However, they found an inverse pattern where the normal kidney actually demonstrates increased signal relative to the adjacent tumor [21].

#### Renal Cyst Unroofing

Similar to renal tumors, healthy renal parenchyma would hyper-fluoresce compared to the relatively hypofluorescent avascular cystic wall [22]. In cases where large cysts may distort normal renal anatomy, ICG can be quite useful to help identify resection margins and ensure complete unroofing of a renal cyst. ICG can also help identify multiple cysts in a multicystic condition, or in the setting of a complex loculated cystic structure. Esposito et al. described using ICG in unroofing 4 renal cysts. They found in this small series that ICG allowed for better identification of the cyst and was safe to use; however, it did not show significant outcome benefit when compared to performing the procedure without ICG [18, 22].

#### Ureteral Reconstruction

ICG’s ability for identification of blood flow was also utilized in confirming ureteral perfusion during pediatric ureteral surgeries. Carty et al. reported their series of 3 patients that underwent robotic surgery for congenital ureteral stricture, mid-ureteral polyp disease and distal ureteral polyp disease. ICG was injected post excision of ureteral stricture segment and/or polyp to demonstrate good post-anastomotic blood flow [23].

Likewise, ICG imaging can help identify the ureter intraoperatively, especially during tumor resection or in cases of significant retroperitoneal fibrosis where the ureter may be encased or adhered to surrounding tissue. The use of ICG can help reduce the increased risk of inadvertent damage. It can also facilitate identification of duplicated ureters in duplex kidneys. One commonly described approach to ureteral identification is to insert an open-ended ureteral catheter endoscopically and injecting ICG solution through the catheter [19].

#### Testicular Torsion

Testicular torsion is a surgical emergency; the challenge with testicular torsion lies in the decision-making process of intraoperative orchiectomy, which most often is based on the macroscopic appearance of the testis. ICG has been used to determine post- torsion perfusion and the extent of ischemia, which can ultimately help with the decision of testicular salvage or removal. In a study by Liu and colleagues, eight patients with testicular torsion received ICG imaging intraoperatively [24]. After intravenous injection, three patients had no evidence of testicular blood flow per ICG fluorescence intraoperatively and underwent orchiectomy, while four patients had return of flow and were salvaged. These four patients had viable testes and blood flow one month after surgery based on testicular doppler ultrasound. One patient with no testicular blood flow per ICG imaging but had visual evidence of parenchymal bleeding underwent testicular salvage, but was later found to have no internal blood flow signals by ultrasound after surgery. Wayne et al. performed a similar study wherein they injected ICG before and after torsion reduction in 30 patients. This was utilized as a decision tool intra-operatively to determine if the testes should be removed or not [25].

#### Hypospadias

ICG’s ability to identify arterial blood supply has also been used in hypospadias repair. Paraboschi et al. has shown that ICG can help identify arterial blood supply in a preputial island flap urethroplasty during complex hypospadias repair [26]. Raines et al. also published a pilot study of 14 patients on utilizing ICG in flap repair of hypospadias. They injected ICG at 3 different time points- after degloving, after flap harvest and mobilization and after securing the flap in place and closing of skin. They found that ICG uptake in the flap decreased with increasing manipulation and impacted intra-operative decision making in one of their 14 cases [27]. Specifically, they noted in one case that ICG uptake was mismatched between two ends of the flap, so they opted to rotate the flap that was less perfused away from the anastomosis to reduce the risk of stricture or scar formation. Unfortunately, their follow-up has not been long enough to determine the usefulness of this technology for hypospadias repair.

#### Bladder Exstrophy Repair

Bladder exstrophy repair is challenging and perfusion is key to healing. Kaefer et al. utilized ICG to study the penile perfusion at 4 different stages during the repair of 8 consecutive patients. They noted a 50% reduction in penile blood flow with apposition of the pubic symphysis and those undergoing CPRE experienced additional 33% drop in blood flow, however, at follow up all penises was noted to be symmetric and healthy with no signs of tissue lost [28]. Similarly, Raines et al. utilized ICG to assess bladder plate and penile perfusion during bladder exstrophy closure in a four-month child with the aim of decreasing penile lose due to ischemia. They demonstrated that after taking down inter-symphyseal bands, the glans perfusion went down to 20–30% uptake on the right glans and 40–50% on the left glans [29]. Paraboschi et al. also utilized ICG in their one-stage delayed bladder closure and radical soft- tissue mobilization. ICG was able to help identify vasculature and assess tissue perfusion during their repair. They injected ICG at 6 different time points during their repair. At the end of the case, they were able to visualize good perfusion of their repair and confirm no vascular injury to the surrounding structure with ICG at the end of the case as well [30].

### Future Directions

While ICG has been used in multiple various urologic procedures, many of these studies remain small, single-center evaluations with short term follow up. Future outcomes studies should focus on larger cohort studies which can help validate the safety and efficacy of this tool.

Other potential uses of ICG in children should be considered particularly in the realm of reconstructive urology where vascular perfusion is critical, such as ureteral anastomosis and reimplantation. Additionally, innovative applications can be explored in the neonatal age where aberrant pelvic anatomy and anorectal malformations may be challenging to fully delineate. Recently, the use of ICG has been applied to Hirschsprung’s Disease and other cloacal and anorectal malformations in pediatric surgery [31]. ICG can similarly aid in appreciating abnormal genitourinary findings in congenital conditions where conventional imaging may not be helpful. Finally, in oncology, ICG has the potential to help with sentinel lymph node biopsies and retroperitoneal lymph node dissections, such as treatment of testicular cancer.

## Conclusions

The use of ICG imaging has been well explored in urology and surgery, and it has now been demonstrated to be safe and effective in pediatric surgery. ICG’s ability to illuminate perfusion and qualify complex anatomy has made it useful in a variety of congenital conditions, such as hypospadias, exstrophy and cloacal anomalies. However, given that all published studies have been small and single-center, it is difficult to ascertain whether this technology can be widely applied in all aspects of pediatric urology and at all ages. Additional research is needed to standardize ICG protocols for use in children and identify whether ICG truly benefits surgical outcomes.

## Key References

**Esposito, C., Coppola, V., Del Conte, F., Cerulo, M., Esposito, G., Farina, A., Crocetto, F., Castagnetti, M., Settimi, A., & Escolino, M. (2020). Near-Infrared fluorescence imaging using indocyanine green (ICG): Emerging applications in pediatric urology. Journal of Pediatric Urology, 16(5), 700–707. 10.1016/j.jpurol.2020.07.008.

- One of the largest single-center studies examining use of ICG in a variety of cases. Detailed description of dosage of ICG and how to administer the compound, followed by outcomes data for varicocelectomy, nephrectomy, partial nephrectomy and renal cyst unroofing.

2. *Raines, A., Fernandez, N., Ahn, J., Cain, M., Joyner, B., Kieran, K., Merguerian, P., & Shnorhavorian, M. (2024). Preputial pedicle flap ICG blood flow assessment during proximal hypospadias repair: Development of a standardized protocol. Journal of Pediatric Urology, 20(4), 691.e1-691.e7. 10.1016/j.jpurol.2024.05.015.

- The first study to design a protocol for use in hypospadias. ICG was used at 3 timepoints to assess flow and viability of the preputial flap. This is an interesting and novel method to examine perfusion to the flap that may not be visible to the eye.

3. *Carty, K. N., Hwang, A., Gordon, A., Locke, R., DeMarco, R. T., & Bayne, C. E. (2021). Indocyanine green (ICG) assessment of ureteral perfusion during pediatric robotic surgery. Journal of Pediatric Surgery Case Reports, 74, 102,058. 10.1016/j.epsc.2021.102058.

- Case series of 3 pediatric patients who underwent robotic surgery for ureteral stricture or ureteral polyp disease. They utilized Firefly^®^ technology embedded within the Intuitive da Vinci System to illuminate perfusion of ureteral edges after anastomosis.

4. *Liu, X., Xu, Y., Li, L., & Bai, D. (2023). Evaluation of testicular blood flow during testicular torsion surgery in children using the indocyanine green–guided near-infrared fluorescence imaging technique. Frontiers in Pediatrics, 11. 10.3389/fped.2023.1272659.

- First published case series of 8 patients who had ICG imaging at time of testicular torsion surgery. This study demonstrates utility of having an objective measurement intraoperatively of testicular re-perfusion.

## Data Availability

No datasets were generated or analysed during the current study.
